# From cell counts to cellular interactions: Cu-Cyto and the co-localization index as a spatial framework for the tumor immune microenvironment of rectal cancer

**DOI:** 10.1007/s10147-026-03182-0

**Published:** 2026-09-08

**Authors:** Kimihiro Yamashita, Toru Nagasaka, Tomoki Abe, Yukari Adachi, Ryota Ito, Takaaki Tachibana, Toru Takahashi, Hiroki Kagiyama, Masaki Imai, Takao Tsuneki, Masayuki Ando, Yuna Asano, Kumiko Miyashita, Yasunori Otowa, Naoki Urakawa, Hironobu Goto, Hiroshi Hasegawa, Shingo Kanaji, Mitsugu Fujita, Takeru Matsuda, Ryohei Sasaki, Takumi Fukumoto, Yoshihiro Kakeji

**Affiliations:** 1 Division of Gastrointestinal Surgery, Department of Surgery, Graduate School of Medicine, Kobe University, 7-5-2 Kusunoki-cho, Chuo-ku, Kobe, Hyogo 650-0017 Japan; 2 Division of Biophysics, Department of Health Sciences, Graduate School of Medicine, Kobe University, 7-10-2 Tomogaoka, Suma-ku, Kobe, Hyogo 654-0142 Japan; 3 Association of Medical Artificial Intelligence Curation (AMAIC), Sakae Members Office Building, Room 505, 4-16-8 Sakae, Naka-ku, Nagoya, Aichi 460-0008 Japan; 4 Center for Medical Education and Clinical Training, Faculty of Medicine, Kindai University, 1-14-1 Miharadai, Minami-ku, Sakai, Osaka 590-0197 Japan; 5 Division of Hepato-Biliary and Pancreatic Surgery, Department of Surgery, Graduate School of Medicine, Kobe University, 7-5-2 Kusunoki-cho, Chuo-ku, Kobe, Hyogo 650-0017 Japan; 6 Division of Radiation Oncology, Graduate School of Medicine, Kobe University, 7-5-2 Kusunoki-cho, Chuo-ku, Kobe, Hyogo 650-0017 Japan; 7 Department of Gastroenterological Surgery, Hyogo Cancer Center, 13-70 Kitaoji-cho, Akashi, Hyogo 673-8558 Japan

**Keywords:** Tumor immune microenvironment, Deep learning, Image cytometry, Co-localization index, CD103, Rectal cancer

## Abstract

The tumor immune microenvironment in colorectal cancer has emerged as a major determinant of patient outcome, with tumor-infiltrating lymphocytes, particularly CD8⁺ T cells, mediating antitumor immunity. Standardized density-based metrics such as the Immunoscore have demonstrated prognostic value but cannot capture the spatial interactions among cells that underlie immune–tumor biology. We introduce a deep learning framework that integrates two complementary axes of analysis: Cu-Cyto, a deep learning–based image cytometry platform that detects and classifies approximately twenty cell types from standard immunohistochemistry-stained whole-slide images, using a bit-pattern kernel-filtering algorithm to prevent multi-counting and an off-target labeling strategy for precise nuclear-center localization; and the Co-Localization Index, which converts the classification probabilities and coordinates produced by Cu-Cyto into a single quantitative measure of co-localization between two or three cell types. We illustrate this framework through CD103⁺CD8⁺ tissue-resident memory–like T cells in rectal cancer treated with neoadjuvant chemoradiotherapy: their stromal but not intratumoral density independently predicts relapse-free survival. This divergence, which compartment-aware density alone cannot explain, motivates a metric that captures within-compartment spatial relationships. The Co-Localization Index extends naturally to three-cell interactions, providing a quantitative readout of the tri-cellular biology among CD103⁺CD8⁺ T cells, tumor cells, and stromal components. Prospective validation in independent rectal-cancer cohorts, addressing watch-and-wait organ preservation and adjuvant chemotherapy decisions, will determine whether this spatial-cellular framework translates into routine clinical decision support with potential extension to other solid tumor contexts.

## Introduction

The tumor immune microenvironment (TIME) has emerged as a major determinant of patient outcome in colorectal cancer. Within the TIME, tumor-infiltrating lymphocytes (TILs), particularly CD8⁺ cytotoxic T cells, mediate antitumor immunity and have been consistently associated with improved survival across solid tumors [1, 2]. In rectal cancer treated with neoadjuvant chemoradiotherapy (NACRT) followed by radical surgery, the abundance and spatial distribution of TILs influence both pathological response and long-term recurrence risk [3–5]. Locally advanced rectal cancer is now managed by multiple modalities, including conventional NACRT followed by radical surgery, total neoadjuvant therapy, and organ-preserving watch-and-wait management for clinical complete responders [6–8]. As these options expand, patient stratification to the most appropriate treatment trajectory remains an unmet need that has only grown. Reliable, reproducible quantification of these cells is therefore central to prognostic biomarker development.

Conventional quantification of immune cells in tissue specimens has relied on manual counting under the microscope or simple thresholding of digitized images. These approaches suffer from observer variability, are difficult to standardize across institutions, and reduce the spatial complexity of the TIME to per-area cell densities [9, 10]. Even among experienced pathologists, visual assessment of immune infiltrate suffers from weak inter-observer agreement, a well-recognized limitation that automated quantification aims to address. Multi-institutional comparisons report discordance from the standardized Immunoscore classification in nearly half of evaluated cases [11]. The standardized Immunoscore developed by Pagès and colleagues has demonstrated that compartment-aware density of CD8⁺ and CD3⁺ T cells carries strong prognostic value in colorectal cancer, with subsequent validation in rectal cancer biopsies prior to neoadjuvant treatment [12, 13]. Yet the density framework cannot capture the spatial interactions among cells that may underlie immune–tumor biology.

The convergence of digital pathology, deep learning (DL), and multiplex immunohistochemistry (IHC) has opened a route to higher-resolution, reproducible TIME analysis. Deep learning–based image cytometry (DL-IC) platforms detect, classify, and quantify individual cells across entire slides, supporting both density-based metrics and spatial coordinate–level analyses [14, 15]. The translation from density to spatial interaction in DL-IC, however, has not yet been formally established as a clinical biomarker framework.

**Fig. 1:**
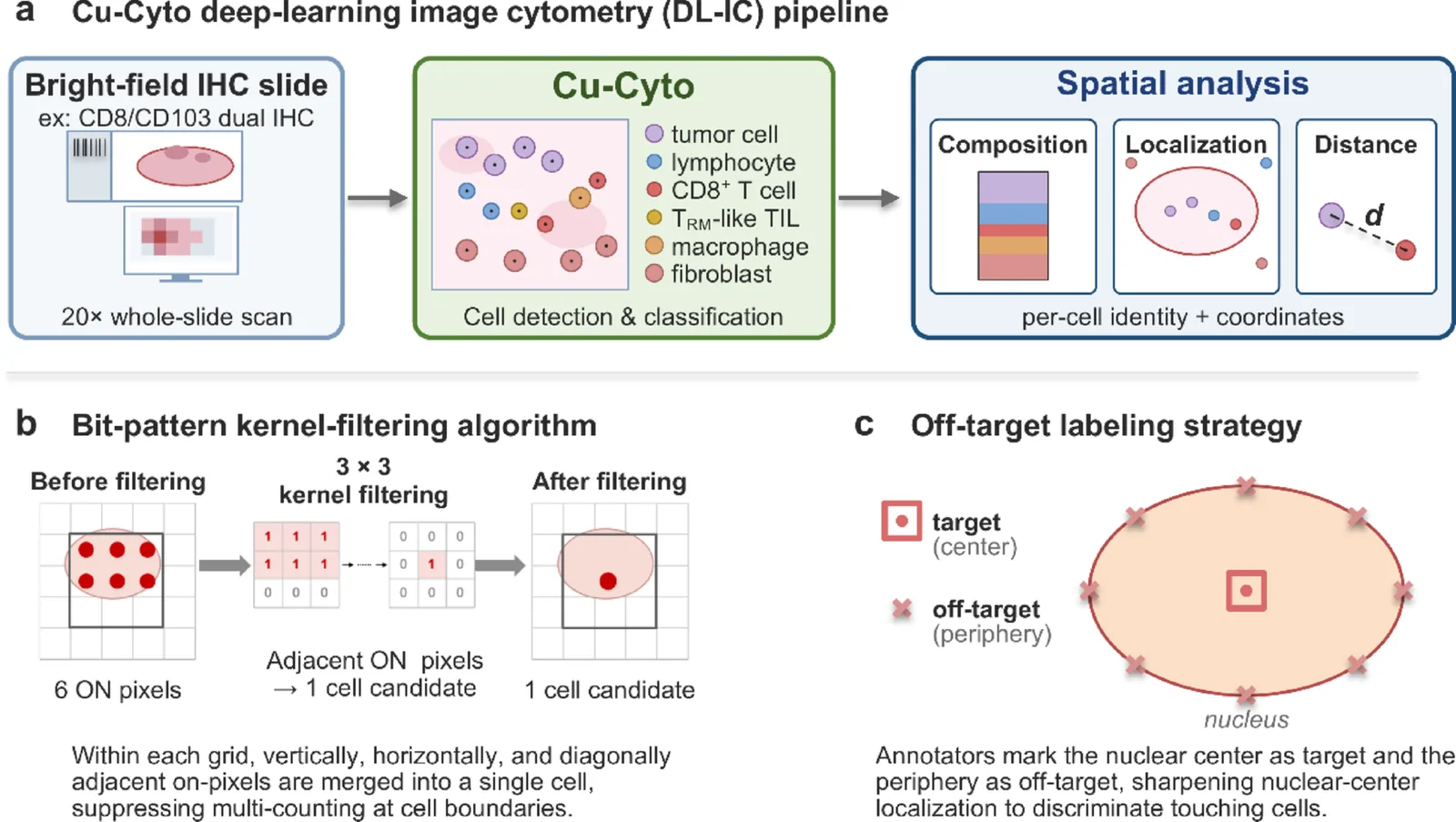
Cu-Cyto deep-learning image cytometry pipeline and its two key algorithmic components. **a** The pipeline converts multiplex immunohistochemistry whole-slide images (left; acquisition at 20× magnification and preprocessing for image normalization) into per-cell identity and coordinates through Cu-Cyto cell detection and classification (center, green highlight), producing three classes of spatial readouts (right): cell-type composition, anatomical localization within tissue compartments, and intercellular distance. Representative detected cell types, color-coded throughout the figure, include tumor cells (purple), lymphocytes (light blue), CD8⁺ T cells (red), CD103⁺CD8⁺ tissue-resident memory–like cells (amber, double-stained for both CD8 and CD103), macrophages (orange), and fibroblasts (pink); the classifier supports approximately twenty cell types in total. **b** The bit-pattern kernel-filtering algorithm partitions whole-slide images into an 18.3 × 18.3 μm grid and merges vertically, horizontally, and diagonally adjacent on-pixels into a single cell, suppressing multi-counting at indistinct or overlapping cell boundaries. **c** The off-target labeling strategy marks the nuclear center (□) as the classification target and the nuclear periphery (×) as off-target during training, forcing the network to learn precise nuclear-center localization and thereby improving discrimination of touching and overlapping cells

**Fig. 2:**
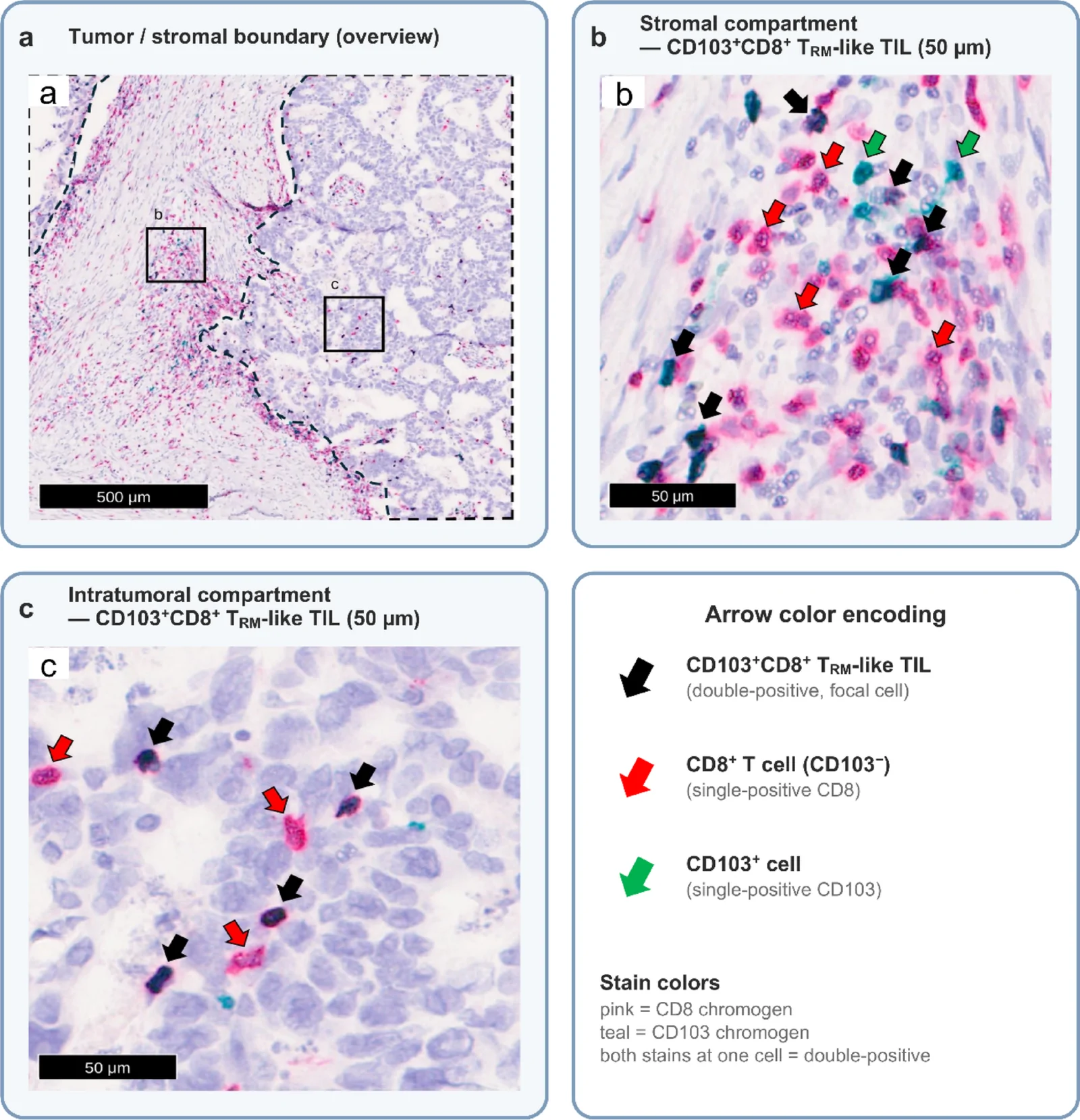
Representative multiplex immunohistochemistry of CD103⁺CD8⁺ tissue-resident memory–like tumor-infiltrating lymphocytes (T_RM_-like TIL) in rectal cancer, comparing intratumoral and stromal (peritumoral) compartments. **a** Low-magnification overview of a rectal-cancer specimen showing the tumor / stromal boundary (dashed line). The stromal (peritumoral) compartment occupies the upper-left half and the intratumoral compartment occupies the lower-right half; boxed areas labeled *b* and *c* indicate the high-magnification fields shown in the corresponding panels. Scale bar, 500 μm. **b** High-magnification view of the stromal compartment showing dense T cell infiltration. **c** High-magnification view of the intratumoral compartment, with comparatively sparse T cell infiltration. In **b** and **c**, arrows mark cells of interest: black, CD103⁺CD8⁺ T_RM_-like TIL (double-positive, focal subset); red, CD8⁺ T cells single-positive for CD8 (CD103⁻CD8⁺); green, single-positive CD103⁺ cells. Pink chromogen marks CD8 expression and teal chromogen marks CD103 expression; cells expressing both stains at one cell are scored as CD103⁺CD8⁺ T_RM_-like TIL. Scale bars, 50 μm. Adapted from Abe et al. [19], with permission

**Fig. 3:**
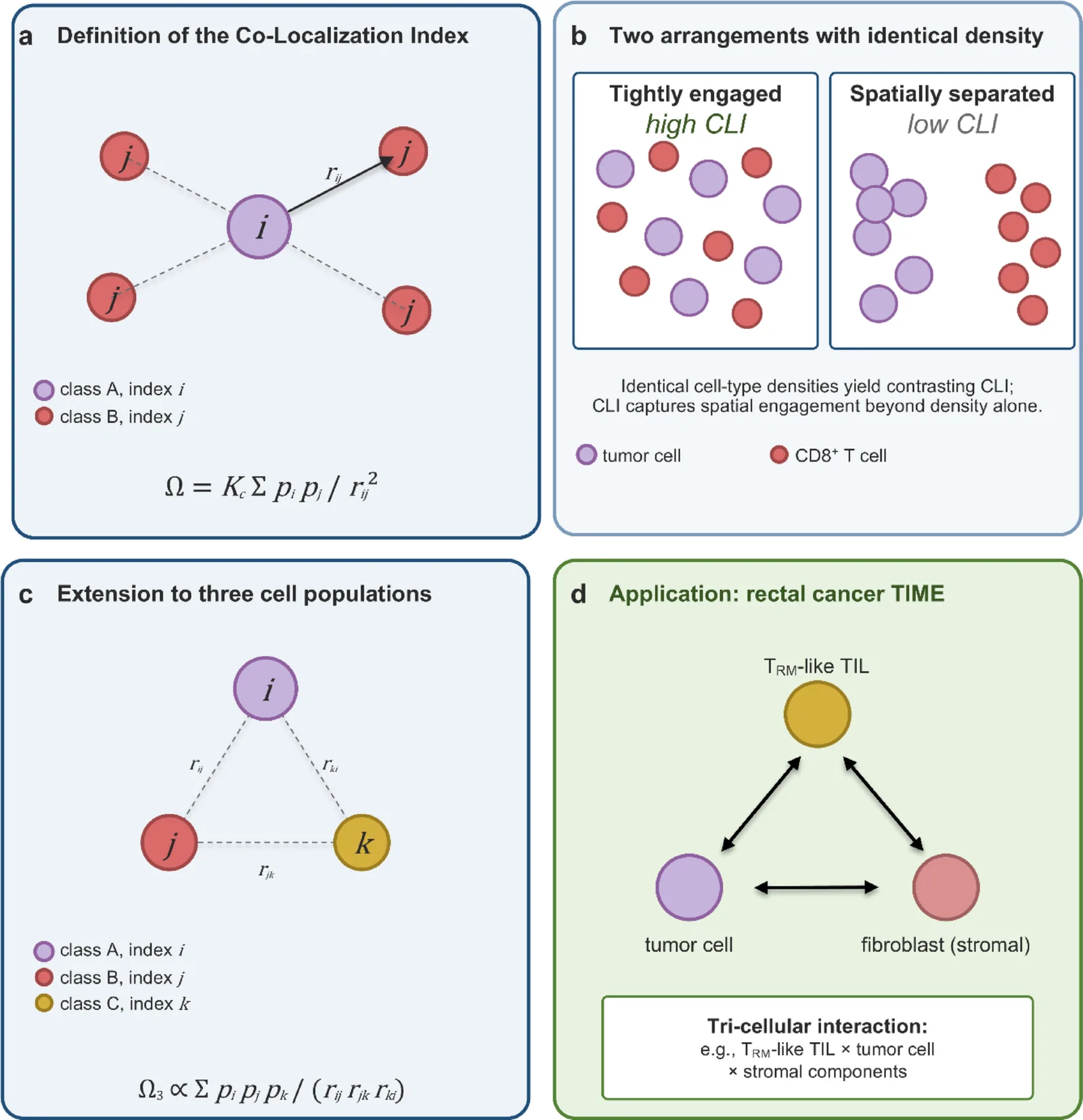
Co-Localization Index (CLI): definition, behavior, extension, and application to the tumor immune microenvironment of rectal cancer. **a** Definition of CLI for two cell populations. For each cell *i* of cell type A (tumor cell, purple) and each cell *j* of cell type B (CD8⁺ T cell, red), CLI is computed as Ω = *K*_c Σ *p*_i *p*_j / *r*_ij², where *K*_c is a normalization coefficient, *p*_i and *p*_j are probability weights, and *r*_ij is the Euclidean distance between cells *i* and *j*. Pairwise inverse-square weighting renders CLI sensitive to spatial engagement rather than to overall density. **b** Two cell arrangements with identical cell-type densities can yield contrasting CLI values: tightly engaged tumor and CD8⁺ T cells (left) yield high CLI, whereas spatially separated populations (right) yield low CLI despite the same per-cell-type density. **c** Extension to three cell populations: the three-cell CLI considers triplets (*i*, *j*, *k*) drawn from cell types A (purple), B (red), and C (amber), with Ω₃ ∝ Σ *p*_i *p*_j *p*_k / (*r*_ij *r*_jk *r*_ki); the proportional sign (∝) absorbs a normalization coefficient analogous to K_c in a. Triplet inverse-product weighting quantifies tri-cellular co-engagement. **d** Application to the tumor immune microenvironment of rectal cancer. The CD103⁺CD8⁺ T_RM_-like tumor-infiltrating lymphocyte (amber, double-stained for both CD8 and CD103) participates in a tri-cellular interaction with tumor cells (purple) and fibroblasts (pink, stromal component); three-cell CLI provides a quantitative framework to assess this engagement

**Fig. 4:**
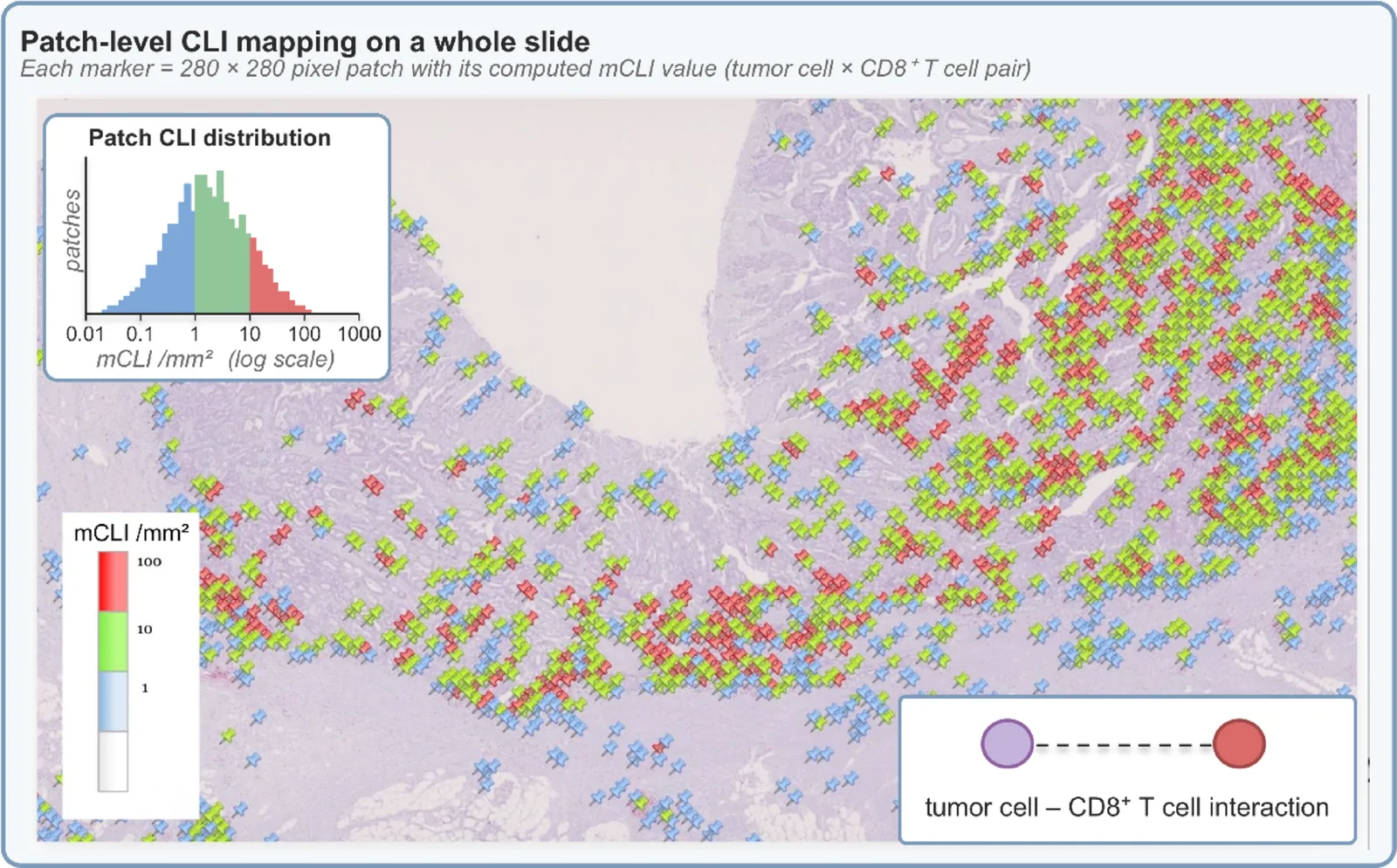
Whole-slide visualization of patch-level Co-Localization Index in rectal cancer. Each 280 × 280 pixel patch within the whole-slide image is assigned a CLI value for the tumor cell × CD8⁺ T cell pair, and patches are color-coded by tier (blue, ~ 1 mCLI/mm²; green, ~ 10 mCLI/mm²; red, ~ 100 mCLI/mm²). The inset histogram (upper left) summarizes the distribution of patch CLI values across the slide on a log scale spanning approximately five decades (0.01–1000 mCLI/mm²); the distribution is approximately log-normal, indicating that most patches carry low or undetectable CLI while a minority concentrate the measurable interaction signal. The legend (lower right) identifies the cell-type pair under analysis (tumor cell, purple; CD8⁺ T cell, red); mCLI denotes milli-CLI (×10⁻³ CLI); CLI is expressed per unit tissue area (mCLI/mm²)

This review is organized around two complementary axes of TIME analysis and how they connect. First, Cu-Cyto, a DL-IC platform, provides cell-level identification and quantification. Whereas many existing DL-IC approaches resolve a more limited predefined set of cell types from a single staining input [14, 15], Cu-Cyto can determine more than twenty cell types within a single pipeline directly from standard bright-field immunohistochemistry, with high classification accuracy for a subset of these types [16]. Its cell detection and counting rely on two algorithmic innovations: a bit-pattern kernel-filtering algorithm to avoid multi-counted cells and an off-target labeling scheme that anchors each detection to the nuclear center [16, 17] (Fig. 1). Second, the Co-Localization Index (CLI), built on the classification probabilities produced by Cu-Cyto, quantifies two- and three-cell interactions in tissue space [18] (Fig. 3). As a worked example, we follow CD103⁺CD8⁺ tissue-resident memory (T_RM_)–like T cells in rectal cancer after NACRT (Fig. 2), a population that serves as a reproducible AI-derived candidate prognostic biomarker. Together, the two axes support integrated whole-slide prognostic analysis in rectal cancer and, in principle, other tumor types (Fig. 4).

### Cu-Cyto: a deep learning platform for cell-level quantification

Cu-Cyto is a deep learning–based image cytometry (DL-IC) platform designed to identify and quantify diverse cell populations from whole-slide images obtained from multiplex immunohistochemistry (IHC) or hematoxylin (H) staining (Fig. 1a). The pipeline integrates four stages: (i) image acquisition with a high-resolution digital scanner; (ii) preprocessing for image normalization; (iii) cell detection and classification using convolutional and residual neural networks; and (iv) spatial analysis of the detected cells. In the rectal-cancer implementation illustrated here, the immunohistochemistry consisted of dual chromogenic staining with anti-CD8 and anti-CD103 antibodies. This marker set directly distinguishes CD103⁺CD8⁺, CD103⁻CD8⁺, and CD103⁺CD8⁻ immunophenotypes, but Cu-Cyto is not restricted to this antibody configuration. The approximately twenty cell types are not defined by one antibody per cell type; rather, in the present implementation, the classifier resolves them from the dual-stain information together with morphology and tissue context, with high classification accuracy for a subset of cell types. Because most of these types are distinguished by morphology and tissue context rather than by antibody staining, the number of resolvable types is not limited by the number of markers. Detailed antibody clones and staining protocols are provided in refs [16, 19]. Cu-Cyto’s design supports TIME analysis along three analytical axes simultaneously within each specimen: *cell composition* (which populations are present and in what proportions, including tumor cells, immune subsets, and stromal components), *spatial localization* (where each cell sits within the tissue, including its anatomical compartment), and *intercellular distance* (how cells are arranged with respect to one another). Because identification and coordinates are produced together for every detected cell, the platform extends from compartment-specific density counts to measurements of cell proximity, neighborhood composition, and spatial patterns among multiple cell types. The classifier can be trained to recognize additional cell populations, as illustrated by its application to CD103⁺CD8⁺ T cells in rectal cancer.

Two algorithmic innovations support Cu-Cyto’s accuracy. The bit-pattern kernel-filtering algorithm addresses multi-counting by partitioning whole-slide images into an 18.3 × 18.3 μm grid and iteratively merging adjacent cell-candidate pixels through kernel matching, suppressing over-counting at indistinct cell boundaries or in densely packed areas [16] (Fig. 1b). The on/off value denotes detected cell presence at each grid point, not a binarized marker-protein level. Accordingly, no cutoff value for marker-protein amount is defined or required in this kernel-filtering step. The off-target labeling strategy sharpens nuclear localization during training: annotators place a pin at each nuclear center while peripheral nuclear regions are explicitly labeled as off-target, forcing the network to learn nuclear-center localization with high precision and thereby improving discrimination between touching and overlapping cells [17] (Fig. 1c).

Beyond these algorithmic features, Cu-Cyto’s classifier is refined through iterative training across successive cohorts of annotated specimens, with each round expanding the diversity of tissue contexts, staining patterns, and rare cell phenotypes that the classifier encounters. Cell-type annotation is performed by trained annotators with pathologist review, and class imbalance is mitigated by oversampling under-represented populations such as CD103⁺CD8⁺ T_RM_-like TILs. This iterative, review-coupled training is a core operating principle of Cu-Cyto rather than a one-time step: each cycle of annotation, pathologist review, and retraining progressively broadens the range of tissues and cell types the platform can reliably resolve.

The cell-classification performance of Cu-Cyto has been evaluated using F1 scores at the grid level (18.3 × 18.3 μm). The F1 score is the harmonic mean of precision and recall, ranging from 0 to 1 with higher values indicating better agreement with reference annotations. With progressive expansion of the training dataset, F1 scores improved across all evaluated cell types and reached 0.911 for CD8⁺ T cells, 0.889 for lymphocytes, and 0.589 for adenocarcinoma cells. The lower adenocarcinoma value reflects their morphological diversity across histological subtypes and the absence of specific immunohistochemistry markers. In line with this, immunohistochemically stained cells consistently outperformed non-immunostained populations, reflecting the contribution of immunohistochemistry to classification accuracy [16]. Iterative dataset expansion continues to improve classification performance across cell types, including for adenocarcinoma cells (unpublished observations).

Two practical features support the use of Cu-Cyto for biomarker development at scale. First, operating on standard bright-field immunohistochemistry rather than specialized multiplex imaging platforms keeps per-specimen cost low and aligns with routine clinical pathology workflows, allowing analysis to scale to the large cohorts needed to relate spatial features to long-term outcomes and to diverse clinicopathological variables. Second, the platform is configured to identify approximately twenty cell types relevant to the TIME [16], including adenocarcinoma cells, glandular epithelial cells, stromal cells, lymphocytes, macrophages, and CD8⁺ T cell subsets such as CD103⁺CD8⁺ T_RM_-like TILs.

### CD103⁺CD8⁺ T cells in rectal cancer: reproducible deep learning–based quantification and a compartment-specific prognostic association

T_RM_-like tumor-infiltrating lymphocytes have emerged as a major focus in tumor immunology, representing a population that establishes durable residence within tumor tissues and mediates rapid, local anti-tumor responses [20]. CD103⁺CD8⁺ T cells represent a distinct T_RM_-like subset of cytotoxic T lymphocytes that infiltrate tumor tissues and engage epithelial cells through E-cadherin–mediated interactions. The CD103 integrin (αE chain) binds E-cadherin on epithelial tumor cells, anchoring these T cells in direct contact with their targets. This structural feature distinguishes CD103⁺CD8⁺ T_RM_-like TILs from recirculating T cells and supports their proposed role as local antitumor effectors.

Across multiple solid tumors including colorectal cancer, abundance of CD103⁺CD8⁺ T cells has been associated with improved patient outcomes [21–23]. Accumulation of CD103⁺CD8⁺ T_RM_-like TILs has also been linked to favorable responses to immune checkpoint blockade in melanoma, non–small cell lung cancer, and breast cancer [24–26], marking this subset as a candidate spatial-immune biomarker. By analogy with tissue-resident memory T cells in non-malignant tissues, their effector function and tissue residency are thought to be sustained within a local niche shaped by stromal cells and stromal cytokine cues [20, 27], placing the biology of T_RM_-like TIL within an inherently three-way relationship among the T cell, the tumor epithelium, and the stromal compartment.

Despite this biological motivation, the integration of CD103⁺CD8⁺ T_RM_-like TIL profiling into NACRT response prediction and organ-preservation decision-making in rectal cancer remains largely unexplored. Cu-Cyto reliably identifies CD103⁺CD8⁺ T cells in immunohistochemically stained rectal cancer specimens (Fig. 2b, c). Two pathologists independently reviewed Cu-Cyto’s cell-type classifications on additional rectal cancer specimens and reached concordant assessments [19], supporting the reproducibility of these density measurements as a prerequisite for AI-derived biomarker adoption [28, 29].

In locally advanced rectal cancer treated with NACRT, stromal CD103⁺CD8⁺ T cell density (Fig. 2b) was identified as an independent predictor of relapse-free survival on multivariate analysis, whereas intratumoral density (Fig. 2c) of the same cell population showed no significant prognostic association [19]. This compartment-specific divergence in prognostic value cannot be explained by cell counts alone, indicating that prognostic information resides in the spatial localization of CD103⁺CD8⁺ T cells, motivating the within-compartment spatial framework introduced below. This compartment-specific pattern differs from primary breast cancer, where intratumoral CD103⁺CD8⁺ T cells, rather than stromal, predict relapse-free survival [30], suggesting that the prognostically informative compartment may vary by cancer type and treatment context.

### Co-Localization Index

Compartment-aware density yields prognostic information in rectal cancer but does not capture the spatial arrangement of cells within a compartment. The Co-Localization Index (CLI) converts deep learning–derived classification probabilities and cell coordinates into a single quantitative measure of spatial co-localization for two cell populations within a defined region of tissue [18] (Fig. 3a).

Within a predefined region (for whole-slide visualization, this region is operationalized as a 280 × 280 pixel patch), CLI sums a weighted contribution from every pair of cells drawn from the two populations. The weight depends on two factors. First, CLI multiplies the two cells’ classification probabilities (p_i × p_j), so uncertain identifications contribute less. Unlike traditional digital pathology, which assigns each cell a single label, modern deep learning classifiers return a probability for each candidate cell type (for example, 80% CD8⁺ T cell and 15% other lymphocyte). Second, the weight is scaled by the inverse square of the distance between the two cells (1/r²), so distant pairs contribute negligibly.

Intuitively, CLI rises when many high-confidence cells of the two populations sit close to each other, and falls when they are sparse, uncertain, or far apart, translating the geometric proximity of cells into a single comparable number.

The value of CLI becomes concrete when two tissues with identical immune cell density differ in spatial arrangement (Fig. 3b). In one, CD8⁺ T cells are mixed in with tumor cells, in direct contact with potential targets; in the other, an equal number of CD8⁺ T cells forms a separate cluster that is spatially distant from the tumor cells. Density alone treats these as equivalent, yet the biological consequences are different. CLI captures the difference as a single number, allowing such configurational variation to be examined quantitatively.

The CLI as defined above operates between two cell populations. Many biologically meaningful interactions, however, involve three cell populations within the same local region (Fig. 3c). The CLI framework extends naturally to this case: each triplet (one cell from each of three populations) contributes a term that is large when the three cells are confidently identified and close to one another. The result is a single number that captures three-cell configurations beyond what the two-population CLI can describe; the same principle extends in theory to four or more cell populations.

Building on this framework, we extend CLI to rectal cancer by training the Cu-Cyto classifier to recognize CD103⁺CD8⁺ T_RM_-like TIL. Recent work indicates that T_RM_-like states are locally specified by antigen reactivity and the surrounding niche [31], shifting the analytical question from cell abundance to local context. Three-cell CLI captures this shift directly: it extracts the spatial relationship among CD103⁺CD8⁺ T_RM_-like TIL, tumor, and stromal components as a single quantitative parameter, enabling systematic quantification of such tri-cellular interactions in the TIME (Fig. 3d).

At the whole-slide level, CLI is computed for each 280 × 280 pixel patch and mapped across the tissue, yielding a patch-level distribution of measurable interaction intensities (Fig. 4). A CLI value can be assigned to every patch without imposing a minimum cell-count threshold: if one of the relevant cell populations is absent, the summation is empty and the CLI value is zero. A positive two-cell CLI requires at least one cell from each of the two populations, whereas a positive three-cell CLI requires at least one cell from each of the three populations. This property makes CLI well suited to whole-slide mapping. Computing CLI for each patch reveals uneven interaction within a single specimen: some regions show dense tumor–immune engagement, while others show little measurable interaction. This view supports both whole-slide prognosis and detailed mapping of immune architecture.

Recent spatial analyses in colorectal cancer support this analytical shift to multi-cellular organization [32–34]. Validation in an expanded, multi-institutional cohort is being considered and will be reported separately.

### Current applications and future directions

Within the AI biomarker landscape for clinical oncology, Cu-Cyto and CLI occupy a distinct position. Immunoscore has established compartment-aware cell density as a reproducible prognostic biomarker in colorectal cancer, yet density discards the spatial architecture in which immune–tumor interactions occur [12]. Whole-image AI approaches, conversely, capture global textural patterns but lack cellular identity and spatial resolution. Cu-Cyto identifies individual cells with their anatomical coordinates, and CLI converts those coordinates into quantitative measures of spatial interaction between two or three cell types, an axis that neither density-based metrics nor whole-image AI provides on its own.

Because Cu-Cyto operates on standard bright-field immunohistochemistry rather than a dedicated acquisition platform, it avoids the specialized instrumentation and substantial per-specimen cost of high-plex spatial methods such as CODEX, MIBI, or Xenium, supporting routine clinical deployment. This comparison concerns deployment feasibility (cost, instrumentation, and routine staining) rather than analytical performance: the two operate at different stages of the workflow and are complementary, and high-plex methods retain advantages in simultaneous marker density and subcellular resolution. CLI is methodologically agnostic to the underlying imaging platform and could in principle be applied to coordinates derived from any cell-level technology. A recent framework for evaluating AI-derived biomarkers in oncology distinguishes gradations of novelty [28]; within it, CLI represents a novel spatial readout with no established equivalent that is at present a candidate rather than a validated biomarker.

The most immediate clinical application is prognostic stratification in rectal cancer after NACRT, where validation of CLI in an expanded cohort is being considered. Concrete scenarios under investigation include stratification of complete responders to NACRT for watch-and-wait organ preservation and identification of patients most likely to benefit from adjuvant chemotherapy. Both applications use the same Cu-Cyto–CLI framework but sample tissue at different moments. The watch-and-wait setting applies CLI to pre-NACRT biopsy specimens to predict durable complete response, since a successful response may leave no measurable tumor–immune interactions in post-treatment tissue. The adjuvant setting applies CLI to post-NACRT resection specimens to flag patients at elevated recurrence risk despite a favorable pathological response. Both decisions hinge on calibrating CLI thresholds against survival endpoints in prospectively followed cohorts. Extension to other solid tumors is a potential further step, as the CLI framework can in principle be applied across cancer types. Beyond clinical use, the same outputs support discovery of spatial patterns among multiple cell types, mechanistic studies of T_RM_-like TIL niche biology [20], and quantitative assessment of treatment-induced changes in the spatial immune landscape.

Clinical AI in oncology is moving toward two-stage pipelines: one stage extracts structured biological information from raw data, and a downstream stage uses that information for clinical prediction. Recent work in colorectal cancer histology applies this design at scale, pairing a deep-learning feature extractor with a transformer-based prediction module across more than 13,000 patients [35]. Cu-Cyto extracts cells and their locations, and CLI derives a spatial interaction metric from these. Together they form the first stage of such a pipeline. The matching downstream module that converts these spatial features into the relevant clinical predictions remains to be developed for rectal cancer after NACRT.

Several caveats apply to the current evidence base: Cu-Cyto’s classification accuracy varies across cell types, the CD103⁺CD8⁺ T_RM_-like TIL findings derive from single-institution analyses pending multi-center validation, and CLI’s inverse-square distance weighting represents one of several biologically plausible formulations. The bit-pattern kernel-filtering algorithm illustrates this trade-off: although it suppresses multi-counting, distinct cells that physically overlap may occasionally be merged and under-counted, a limitation inherent to grid-based cell determination [16]. At the whole-slide level, CLI is mapped on a fixed patch tiling for visualization, so an interaction whose two cells fall in different patches is not counted in either patch. A peripheral margin of each visualization patch is excluded from the patch-level calculation to limit boundary effects, although these have not been formally quantified. If cross-patch interactions are of primary interest, CLI can instead be computed over larger regions of interest or with overlapping/sliding windows. Practical adoption will require analytical validation across institutions with diverse staining protocols and scanners, prospective evidence of clinical utility, and integration of the spatial readout into pathology workflows that are still optimized for slide-level interpretation. Achieving this in rectal cancer after NACRT will also require overcoming persistent barriers to AI pathology adoption [29].

## Conclusion

Cu-Cyto and CLI together establish a deep learning–based framework for moving beyond density toward spatial structure in the TIME. By preserving cell-level identity and coordinates and translating them into quantitative measures of spatial interaction, including the multi-cell configurations central to T_RM_-like TIL biology in rectal cancer after NACRT, the framework occupies a distinct position alongside density-based prognostic biomarkers and whole-image AI approaches. Positioning Cu-Cyto and CLI as a spatial-cellular extractor within emerging two-stage AI pipelines indicates how this framework may contribute to integrated prognostic systems for the TIME of rectal cancer and other solid tumor contexts. Whether this spatial-cellular framework translates into clinical decision support will ultimately be determined by demonstrating decision-making benefit in routine rectal cancer practice.

## Data Availability

Data sharing is not applicable to this article as no new datasets were generated or analyzed during the current study. The Cu-Cyto deep learning platform and the Co-Localization Index framework are described in detail in references [16] and [18], respectively.
